# Protection of LiFePO_4_ against Moisture

**DOI:** 10.3390/ma13040942

**Published:** 2020-02-20

**Authors:** Nicolas Delaporte, Michel L. Trudeau, Daniel Bélanger, Karim Zaghib

**Affiliations:** 1 Département de Chimie, Université du Québec à Montréal, Case Postale 8888, succursale Centre-Ville, Montréal, QC H3C 3P8, Canada; delaporte.nicolas@hydro.qc.ca; 2 Institut de Recherche d’Hydro-Québec (IREQ), 1800 Boulevard Lionel Boulet, Varennes, QC J3X 1S1, Canada; trudeau.michel@hydro.qc.ca

**Keywords:** surface modification, trifluoromethylaniline, diazonium, grafting, hydrophobic powder, olivine, cathode, Li-ion

## Abstract

In this study, a carbon-coated LiFePO_4_ (LFP/C) powder was chemically grafted with trifluoromethylphenyl groups in order to increase its hydrophobicity and to protect it from moisture. The modification was carried out by the spontaneous reduction of in situ generated 4-trifluoromethylphenyl ions produced by the diazotization of 4-trifluoromethylaniline. X-ray photoelectron spectroscopy was used to analyze the surface organic species of the modified powder. The hydrophobic properties of the modified powder were investigated by carrying out its water contact angle measurements. The presence of the trifluoromethylphenyl groups on the carbon-coated LiFePO_4_ powder increased its stability in deionized water and reduced its iron dissolution in the electrolyte used for assembling the battery. The thermogravimetric and inductively coupled plasma atomic emission spectroscopy analyses revealed that 0.2–0.3 wt.% Li was deinserted during grafting and that the loading of the grafted molecules varied from 0.5 to 0.8 wt.% depending on the reaction conditions. Interestingly, the electrochemical performance of the modified LFP/C was not adversely affected by the presence of the trifluoromethylphenyl groups on the carbon surface. The chemical relithiation of the grafted samples was carried out using LiI as the reducing agent and the lithium source in order to obtain fully lithiated grafted powders.

## 1. Introduction

LiFePO_4_ is one of the most promising cathode materials for lithium-ion batteries [1]. Nonetheless, LiFePO_4_-based batteries exhibit electrolyte decomposition, especially in the presence of traces of water or other protic species, which leads to metal dissolution and the formation of a passivation layer [2,3,4]. Furthermore, the aging and partial oxidation of LiFePO_4_ upon exposure to ambient air and humidity deteriorate its electrochemical performance [5,6,7]. These problems can be mitigated by storing LiFePO_4_ in costly special packaging under inert gas. Recently, in order to improve the stability of LiFePO_4_, the surface modification of a carbon-coated LiFePO_4_ (LFP/C) powder with hydrophobic groups has been reported [8]. In fact, the functionalization of copper and glassy carbon electrodes with 4-trifluoromethylphenyl groups increases their water contact angles, thus increasing their hydrophobicity [9,10]. The modification of carbon powder (e.g., Vulcan XC72 or carbon black), typically used for the preparation of Pt/C active layers of proton-exchange membrane fuel cells (PEMFC), is carried out by grafting it with 4-trifluoromethylphenyl groups [9,11]. This modification enables the production of gas diffusion electrodes with a precisely controlled degree of hydrophobicity.

Substituting X-phenyl-NH_2_ (ex: X = -SO_3_H, -CF_3_) aromatic amines in the presence of a nitrosating agent leads to the formation of the corresponding diazonium cation X-phenyl-N_2_^+^. The reduction of diazonium salts has been widely used for functionalizing surfaces [12,13,14,15,16,17]. This functionalization can not only be achieved electrochemically, but also by the chemical reaction of the surface with in situ generated diazonium salts [18,19,20]. This chemical grafting method leads to the attachment of different substituted aryl groups with the formation of a strong C-C bond [21,22].

Diazonium chemistry has been employed for the modification of Li-ion battery materials. This modification procedure enables the stabilization of carbon anodes by the formation of lithium benzoate [23] and grafted nitrophenyl layers [24]. Silicon anodes [25,26,27,28,29] modified with organic molecules derived from diazonium ion precursors exhibit superior cycling stability. The grafting of silicon anodes with polyacrylic acid via radical polymerization also improves their mechanical and electrochemical properties [29]. Positive electrode materials such as Li_1.1_V_3_O_8_ are functionalized with nitrophenyl groups [30], and redox molecules are attached to powders to assist the insertion of Li^+^ ions [31]. In our previous studies, we used this method to modify LFP/C electrode materials and grafted aminophenyl [32], bromophenyl [32], and benzene-trifluoromethanesulfonylimide [33] moieties on their carbon surfaces. The modified electrode material showed improved electrochemical performance during galvanostatic cycling at high charge/discharge current densities. The modification of LFP/C electrode materials with benzene-trifluoromethanesulfonylimide improves their wettability to conventional carbonate-based electrolytes [33].

**Scheme 1:**
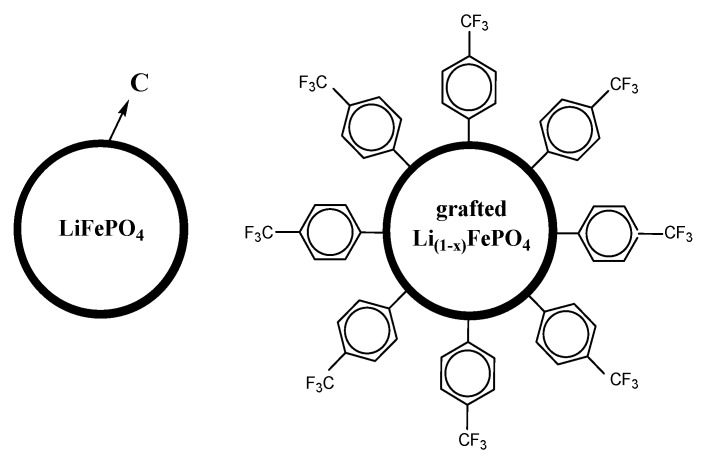
Schematic of the unmodified (**left**) and modified (**right**) carbon-coated LiFePO_4_ (LFP/C) powders.

Herein, we grafted trifluoromethylphenyl moieties onto the carbon surface of LFP/C via covalent carbon-carbon bonds. The surface modification of LFP/C occurred by the reduction of in situ generated 4-trifluoromethylphenyl ions (CF_3_-phenyl-N_2_^+^) produced by the diazotization 4-trifluoromethylaniline (CF_3_-phenyl-NH_2_) in the solution, as illustrated in Scheme 1. The modified material was characterized by chemical analysis (ICP-AES), thermogravimetric analysis (TGA), and X-ray photoelectron spectroscopy (XPS). The chemical stabilities of LiFePO_4_ in the presence of water and in the electrolyte used in the battery were also investigated. The functionalized LFP/C consisted of 0.5–0.8 wt.% of the grafted groups depending on the reaction and delithiation conditions. Finally, to validate the impact of surface modification on the electrochemical properties of LFP/C material, basic electrochemical tests were realized. The electrochemical performance of the modified LFP/C was investigated and was found to be highly dependent on the amount of trifluoromethylphenyl groups attached on the carbon surface. The chemical relithiation of oxidized modified LFP/C was carried out using LiI as the reducing agent and lithium source and was confirmed by X-ray diffraction (XRD) experiments.

## 2. Experimental Section

### 2.1. Surface Modification of LFP/C

The carbon-coated LiFePO_4_ powder containing 2–3 wt.% of carbon was provided by Hydro-Québec, Montréal, Canada. The surface modification of LFP/C was carried out according to a procedure used by us in our previous studies [32,33]. Briefly, 1.5 g of LFP/C was dispersed in 50 mL of acetonitrile (HPLC grade) followed by the direct addition of various amounts of 4-trifluoromethylaniline (2–8 mmol, CF_3_-phenyl-NH_2_) (Sigma-Aldrich) (Table 1). After the addition of an accurate volume corresponding to 3 equivalents of tert-butyl nitrite (Sigma-Aldrich, Oakville, Canada), the mixture was stirred overnight at 25 °C. After the first decantation and removal of the liquid part, the powder was dispersed in acetonitrile, and the mixture was vacuum filtered using a Büchner assembly and a nylon filter (pore size ~0.47 µm). The modified powder was washed several times with acetonitrile and dimethylformamide and then rinsed with acetone followed by vacuum drying overnight at 70 °C. The modified powders were labeled as TFN1, TFN2, TFN3, and TFN4 depending on the precursor (amine and tert-butyl nitrite) concentrations.

### 2.2. Chemical Lithiation of Partially Oxidized LFP/C Powders

After the modification, the LFP/C powders exhibited partial oxidation along with the loss of a small amount of lithium [34]. The relithiation of the grafted-LFP/C samples was realized as follows: 1.5 g of the modified powder were dispersed in a 0.5 M solution of LiI (Li:Fe = 6:1) in dry acetonitrile. The reaction was carried out for 24 h under Ar flux to avoid the oxidation of LiI. The solution was then filtered, washed several times with dry acetonitrile, and dried under vacuum at 70 °C for at least 12 h.

### 2.3. Preparation of the Samples for Iron Dissolution Experiments

The volume of electrolyte used for assembling the button cells was about 150 µL. Hence, its chemical analysis could not be carried out immediately after the cycling. However, the analysis of the solvent was essential for detecting the dissolved metals [4], degraded species, or water generated by the transesterification of alkyl carbonates [35]. In order to estimate the quantity of iron dissolved in the electrolyte, a simple procedure was developed. About 50 mg of the unmodified- and modified-LFP/C (sample TFN4) were dispersed in 10 mL of 1 M LiPF_6_ in ethylene carbonate (EC)/diethyl carbonate (DEC)/dimethyl carbonate (DMC) (1:1:1 by vol.) electrolyte for 1 month. The solutions were stored in a sealed glass vessel under argon. After 1 month, each sample was centrifuged to separate the powder and solvent. Then, 4 mL of the liquid part were dissolved in 20 mL of 18 M nitric acid, and the solution was boiled until a transparent liquid was obtained. The rest of the solution was diluted to 50 mL with 5% HNO_3_.

### 2.4. Characterization

The polycrystalline samples were characterized by XRD using a Philips X’Pert diffractometer (Amsterdam, The Netherlands) in the θ–2θ mode with Cu Kα_1_,α_2_ radiation (λ_1_ = 1.5405 Å, λ_2_ = 1.5443 Å) and a monochromator to avoid the presence of K_β_ radiation. The data were collected over the 2θ range of 15–60° in steps of 0.03° and an integration time of 5 s per step using an X’Celerator detector.

TGA analyses (TA Instruments, New Castle, DE, USA) were carried out with a TGA Q500/Discovery MS model under a constant air flow of 90 mL·min^−1^ over the temperature range of 30–750 °C at a heating rate of 10 °C·min^−1^. The mass spectrometry (MS) of the samples was carried out during the TGA measurements, and all the fragments over the 30–300 m/z range were analyzed.

The hydrophobicity of pristine and modified LFP/C samples was evaluated by depositing 30 µL of water on the corresponding composite electrode (see below). The water contact angles of the samples were measured using a device built in-house. The device consisted of a lens to magnify the water droplet. The contact angles (°) of the samples were measured from the magnified numerical image obtained using ImageJ software.

The XPS (Physical Electronics (PHI), Chanhassen, MN, USA) analysis of the samples was carried out on a PHI Versa Probe III spectrometer using a monochromatic 25 W Al K_α_ radiation (*hν* = 1486.6 eV) focused on the region with a diameter of 100 µm. All three samples were pressed to form thin pellets. The pass energies for survey and high-resolution spectra were 280 and 69 eV, respectively. All the spectra were referenced to C-C or C-H at 284.8 eV. The semi-quantitative atomic concentration was determined through Multipak software using the area of the individual peak and PHI relative sensitivity factor.

The lithium content of the unmodified and modified LFP/C powders was determined using ICP-AES (Thermo Jarrell Ash Trace Scan, Waltham, MA, USA). Acidic digestion was carried out to prepare the samples. Typically, 40 mg of the LFP/C powder were added to 30 mL of 18 M nitric acid, and the solution was boiled until transparency. After dilution to 100 mL with 5% HNO_3_, the solution was injected in the plasma. The lithium contents of the samples were calculated from the calibration curve of various Li concentrations prepared using a lithium atomic spectroscopy standard solution Fluka ([Li] = 1000 ppm, prepared with LiNO_3_ and HNO_3_ 0.5 M). The samples prepared for the iron dissolution experiments were injected into an air/acetylene flame. After 10 s of stabilization, a measurement was recorded. A total of three measurements (5 s each) for iron absorbance at the wavelength of 372 nm were recorded. The iron contents of the samples were obtained from the calibration curve of various Fe concentrations prepared using an iron standard solution (Sigma-Aldrich ([Fe] = 1000 ppm).

### 2.5. Battery Preparation and Electrochemical Testing

The modified powders were mixed with conductive acetylene black and polyvinylidene difluoride (PVDF) in a weight ratio of 80:10:10 using 1-methyl-2-pyrrolidone (Alfa Aesar, 99%) as the solvent. The slurry was cast on an aluminum foil (15 µm) and dried at 70 °C under vacuum for 24 h. The film was cut into circular disks (area = 1.767 cm^2^) having an active mass loading of about 4 mg·cm^−2^ and an electrode density of ~1 g·cm^−3^. The two-electrode electrochemical coin cells were assembled using the Li_4_Ti_5_O_12_ anode (LTO), Celgard^®^-2320 separator, an electrolyte consisting of 1 M LiPF_6_ in ethylene carbonate (EC)/diethyl carbonate (DEC)/dimethyl carbonate (DMC) (1:1:1 by vol.), and the LiFePO_4_/C cathode. The LTO anode provided by Hydro-Québec consisted of 82 wt.% Li_4_Ti_5_O_12_. An electrode mass loading of 5.8 mg·cm^−2^ was used. The cells were assembled in a dry argon-filled glove-box and then controlled with a VMP3 potentiostat. Charge/discharge cycling was carried out in the galvanostatic mode over the potential range of 0.5–2.5 V (vs. LTO) at different current densities after 1h of rest at open circuit voltage (OCV). The current density of 170 mA·g^−1^ corresponded to the 1C rate. For each current density ranging from C/10 to 5C, 10 cycles were recorded within 1 min at OCV before each current density. Electrochemical impedance spectroscopy (EIS) measurements were carried out at an AC amplitude of 10 mV over the frequency range of 200 kHz–0.01 Hz at 2.5 V (vs. LTO). The EIS measurements were carried out after five cycles of charge/discharge at a C/2-rate after applying a constant potential of 2.5 V (vs. LTO) for 4 h.

## 3. Results and Discussion

### 3.1. Characterization of the LFP/C Powders

(a) Thermogravimetric analysis

**Figure 1:**
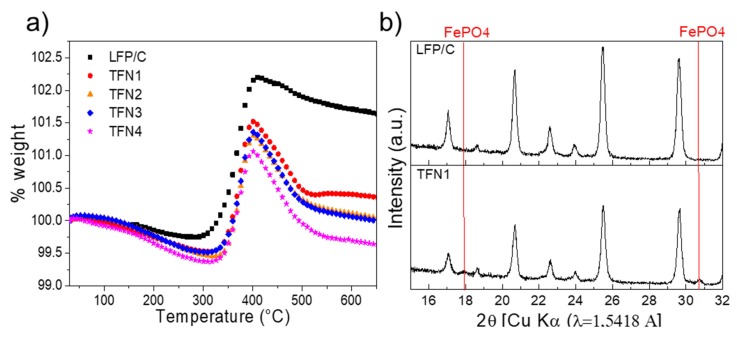
(**a**) TGA curves of the unmodified and modified LFP/C powders recorded in air. (**b**) XRD patterns for pristine LFP/C and TFN1.

Figure 1a shows the TGA results of pristine and modified LFP/C powders with different trifluoromethylphenyl group loadings. The unmodified LFP/C showed a weight gain of 2.5% over the temperature range of 300–400 °C followed by a weight loss of approximately 0.6% over the temperature range of 430–600 °C. The weight gain of LFP/C was caused by the air oxidation of LiFePO_4_ and the formation of Li_3_Fe_2_(PO_4_)_3_ and Fe_2_O_3_ [36,37]. The mass loss of LFP/C could be attributed to the oxidation (burn off) of the carbon coating. The carbon content of the unmodified LFP/C sample was estimated by comparing its TGA curve with that of uncoated LFP as described in [36]. The difference in the weights of the samples after the complete oxidation at 600 °C could be attributed to the amount of carbon present at the surface of the LFP/C particles, which was found to be 2.2 wt.%.

The trifluoromethylphenyl-modified LFP/C powders showed significantly different TGA curves. The oxidation onset temperatures of the trifluoromethylphenyl-modified powders were slightly higher than that of the unmodified LFP/C. A similar trend (less pronounced) has been reported for LFP/C powders modified with bromobenzene [32] and trifluoromethylsulfonimidebenzene groups [33]. Ueda et al. [38] showed that the thermal stability of carbon-coated fluorinated LiFePO_4_ was superior to that of untreated LFP, and the decomposition temperature of fluorinated-LFP was 14 °C higher than that of untreated LFP. Thus, the thermal stability of LFP/C could be improved by carrying out its surface modification. The modified powders exhibited much lower mass gains (325–400 °C) than the unmodified LFP/C powder. The mass gain of the modified powders decreased with an increase in the concentration of the precursors (e.g., amine and tert-butyl nitrite) (see Table 1). This indicated that the oxidation of the LFP powder was more pronounced at high precursor concentrations. This is consistent with the proposed grafting mechanism of LFP/C [34]. The oxidation level of the modified powders was estimated by assuming that the 2.5 wt.% gain for unmodified LFP/C corresponded to 100% oxidation (Table 1) (x in Li_(1-x)_FePO_4_ by TGA). These results were similar to those obtained by chemical analysis of lithium after the acidic digestion of the modified LFP/C powders (x in Li_(1-x)_FePO_4_ by ICP). The oxidation level ranged from 20 to 32%. The higher mass loss of the grafted samples over the temperature range of 400–600 °C than that of the ungrafted sample could be attributed to the presence of the grafted groups in the grafted samples [32,33,34]. Furthermore, as compared to the unmodified LFP/C, the modified LFP/C samples showed a higher mass loss, which increased with an increase in the concentrations of amine and tert-butyl nitrite (Table 1, wt.% of the grafted groups, as revealed by the TGA results).

(b) XRD

The oxidation of the LFP/C powders after grafting was further analyzed by XRD. The XRD pattern of pristine LFP/C is shown in Figure 1b. The diffraction peaks of pristine LFP/C could be indexed to the diffraction peaks corresponding to the Pnmb orthorhombic space group (JCPDS 01-081-1173) [39]. For clarity, magnified XRD patterns (over the 2θ range of 15–32°) were obtained. Figure 1b also shows the XRD pattern of TFN1, which showed additional low intensity peaks at 2θ = 18 and 31° corresponding to delithiated FePO_4_. The presence of FePO_4_ indicated that LFP was oxidized upon grafting. Moreover, the peak intensity of the LFP phase decreased slightly, confirming the existence of a secondary delithiated phase [39]. The intensities of the additional FePO_4_ peaks (relative to those of the LFP peaks) were very low compared to the oxidation levels calculated using the TGA and ICP results. This can be attributed to the nanometric nature of the LFP/C powder, which made it difficult to detect the delithiated phase at low oxidation levels [40,41,42]. Kobayashi et al. showed that the formation of the solid solution was dependent on the size of the LFP particles. For the same oxidation level (x = 0.93 in Li_(1-x)_FePO_4_), the XRD pattern of the powder composed of the smallest particles showed only one phase unlike that of the powders consisting of larger particles [43]. The acquisition mode and powder quality also affected the intensity and resolution of the peaks significantly. Indeed, with a lower oxidation level than that used in this study, LFP/C powders modified with trifluoromethylsulfonimidebenzene groups showed higher delithiated phase contents [33]. Mössbauer spectroscopy can provide a better estimation of the oxidation level of LFP powders [44].

(c) XPS

**Figure 2:**
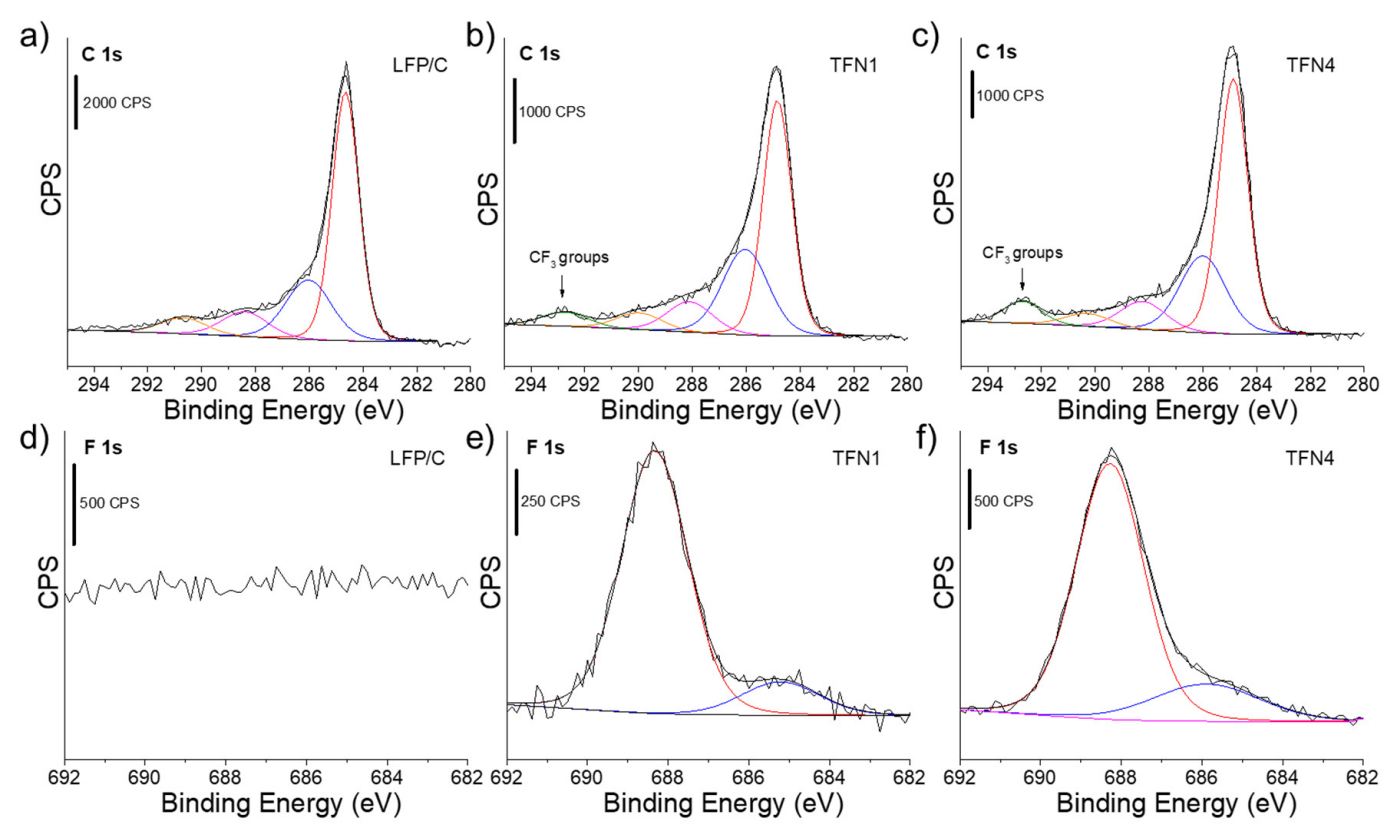
XPS C 1s core level spectra for: (**a**) the unmodified, (**b**) TFN1, and (**c**) TFN4 powders. XPS F 1s core level spectra for: (**d**) the unmodified, (**e**) TFN1, and (**f**) TFN4 powders.

SEM images did not permit putting into evidence surface modification using diazonium chemistry, and as a consequence, X-ray photoelectron spectroscopy was used. SEM images after and before modification using the same nanometric LFP material have been reported in our earlier work [32]. XPS was used to confirm the presence of 4-trifluoromethylphenyl groups on the surface of the modified LFP/C powders. The survey spectra for the modified and pristine LFP/C powders are shown in Figure S1. Figure 2a–c shows the C 1s core level spectra for the LFP/C, TFN1, and TFN4 powders, respectively. In all the spectra, the intense peak located at 284.8 eV could be attributed to the non-functionalized sp^2^ and sp^3^ C atoms from the thin carbon layer around the LFP particles. The weak peaks between 286 and 291.5 eV confirmed the presence of oxygen functionalities such as C−O and C=O bonds at the carbon surface [4,45]. The presence of a large number of oxygen-containing species could be attributed to the use of tert-butyl nitrite for the grafting reaction [34]. However, the slight increase in the intensity of the peak at 286 eV after the modification (see Figure 2b,c) could also be explained by the presence of sp^2^ C−N bonds [46]. These two hypotheses seemed to be confirmed by the atomic concentrations for each sample. As can be observed from Table 2, the samples consisted of large amounts of carbon (attributing to the carbon coating) and oxygen. The presence of a large amount of oxygen could not only be attributed to the oxygen functionalities at the carbon surface, but also to the phosphate of the host material as the surface O:P ratios for LFP/C and TFN1 and TFN4 were approximately four and 4.6, respectively. The surface nitrogen concentrations of the modified powders were slightly higher than that of the unmodified LFP/C sample. Since the grafting reaction required the formation of diazonium ions and the subsequent loss of nitrogen to yield aryl radicals [12], nitrogen could not be detected. However, the presence of nitrogen species on materials modified by diazonium chemistry is well documented and can be attributed to the presence of azo bonds [32,47]. These results provided indirect evidence for the occurrence of grafting at the surface of the LFP/C powders.

Furthermore, the presence of trifluoromethylphenyl groups on the modified-LFP/C surface was clearly confirmed by the presence of the peak at 293 eV (CF_3_ groups) in the C 1s core level spectra of the TFN1 (Figure 2b) and TFN4 (Figure 2c) powders [10,48]. In addition, unlike the F 1s core level spectrum of the LFP/C powder, which only showed background noise (see Figure 2d), the F 1s spectra of TFN1 (Figure 2e) and TFN4 (Figure 2f) showed a strong peak at about 688 eV with an undefined small peak at 685 eV. The peak at 688 eV could be attributed to the covalently bonded fluorine atoms (i.e., CF_3_ groups) [48,49], while that at 685 eV could be attributed to the adsorbed or entrapped fluorine [48]. The fluorine concentrations of the TFN1 and TFN4 powders increased from 3.0 to 5.5 at.% with an increase in the concentration of the reagents (Table 2).

### 3.2. Stability of the LFP/C Powder in Water and Electrolyte

**Figure 3:**
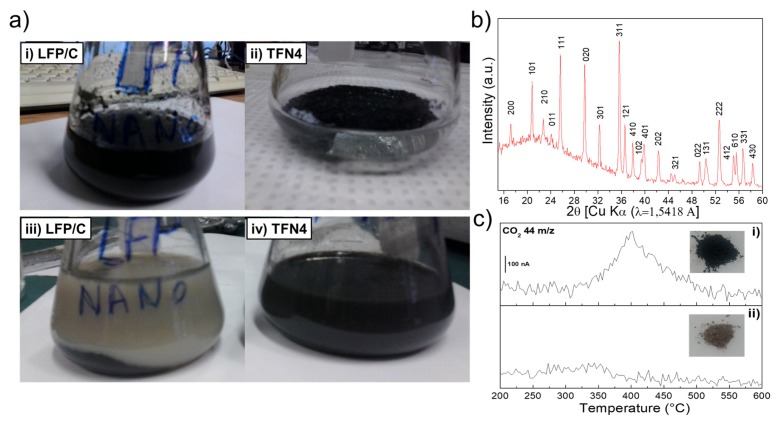
(**a**) Optical photographs of: (**i**) the unmodified and (**ii**) modified LFP/C (TFN4) powders dispersed in water; (**iii**) the unmodified and (**iv**) modified LFP/C (TFN4) powders after 24 h of reaction in water at room temperature. (**b**) XRD pattern of the brown powder obtained after stirring the LFP/C powder in deionized water at room temperature for 24 h. (**c**) Mass spectra of the powders recorded during their TGA measurements and the optical photographs of: (**i**) LFP/C and (**ii**) the brown powder obtained after mixing LFP/C in water for 24 h at room temperature. CO_2_ formed during the decomposition of the powder was detected for a mass/charge ratio (m/z) = 44.

In order to evaluate the hydrophobic properties of the functionalized powder, the unmodified (LFP/C) and modified (TFN4) powders were dispersed in distilled water for 24 h under constant stirring at ambient temperature. Figure 3a (i) shows that initially, LFP/C formed a good dispersion in distilled water unlike the TFN4 sample, which stayed at the surface of water even under vigorous stirring (Figure 3a (ii)). After 24 h of stirring, the modified powder was finally dispersed in water to form a black suspension (Figure 3a (iv)), while LFP/C formed a brown mixture with black LFP/C agglomerates at the bottom, as shown Figure 3a (iii). In addition, a black layer, presumably composed of carbon agglomerates, could be observed at the surface of the LFP/C suspension after the reaction (Figure 3a (iii)) [5]. The filtration and centrifugation of the LFP/C mixture yielded a brown powder. A much larger amount of this powder was obtained when the experiment was carried out at 60 °C for 18 h. The XRD pattern of the brown powder (Figure 3b) showed low intensity peaks with low resolution because a small amount of powder was analyzed. The broad background at around 2θ = 36° could be attributed to the glass support. All the observed peaks could be indexed to those of LFP [39].

TGA coupled with MS was used to analyze the brown powder. The mass spectra of the brown and LFP/C powders are shown in Figure 3c along with their optical images. In the case of LFP/C (Figure 3c (i)), the decomposition of the carbon coating occurred at 350–600 °C in air along with the formation of CO_2_ (m/z = 44). The brown powder (Figure 3c (ii)), on the other hand, showed no significant peak over this temperature range. This indicated that the reaction between water and LFP/C deteriorated the carbon coating of the LFP/C powder, confirming the carbonaceous nature of the black layer observed on the top of water after 24 h of stirring (Figure 3a (iii)). On the other hand, the grafting of hydrophobic groups preserved the carbon layer (no brown powder was recovered even after stirring at 60 °C for 18 h).

The stability of the samples towards oxidation in water was evaluated by estimating the quantity of deinserted lithium (x) using ICP-AES. Table 3 lists the x values for the LFP/C and TFN4 powders in water under different reaction conditions. After the grafting process, the modified LFP/C powder (TFN4) showed slight oxidation because of its x value of 0.28. When the unmodified LFP/C powder was mixed in water, the oxidation of LiFePO_4_ occurred [5,7,50] with an increase of x (Li_(1-x)_FePO_4_/C) from ~0 to 0.34 and 0.29 wt.% when the reaction was carried out for 24 h at room temperature and 18 h at 60 °C, respectively. Grafting with trifluoromethylphenyl groups protected LiFePO_4_ from oxidation. After 24 h of reaction in water, the modified powder lost only 0.02 wt.% of Li (0.34 wt.% for the unmodified LFP/C). The value of x increased to 0.04 when the reaction was carried out at 60 °C for 18 h. The surface functionalization of LFP/C prevented its oxidation and the degradation of the carbon coating.

The powders were exposed to the electrolyte in half-cells. It was found that the modified LFP/C powder showed considerably lower iron dissolution in the electrolyte than the LFP/C powder (see Table S1). Thus, the functionalization of LFP/C with trifluoromethylphenyl groups prevented the dissolution of the active material in the electrolyte, thus increasing the lifespan of the battery.

**Figure 4:**
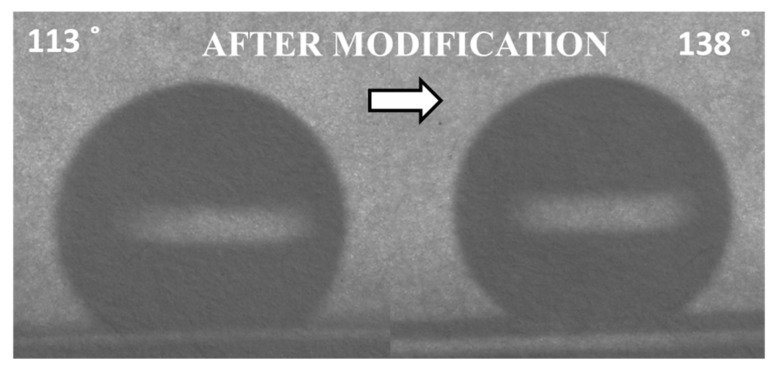
Water contact angle of the unmodified (left, 113°) and modified LFP/C electrodes (right, 138°).

In order to further investigate the hydrophobic properties of the modified powder, the water contact angles of the modified and unmodified electrodes were evaluated. Figure 4 shows the photographs of the unmodified (left) and modified (right) LFP/C electrodes. Because of the presence of PVDF as the binder, the water contact angles of the electrodes (10 wt.%, even the LFP/C cathode) were quite high. However, after the surface modification, the contact angle of LFP/C increased from 113 to 138°, indicating that the modified LFP/C powder was much more hydrophobic than LFP/C. An opposite result was obtained with a water contact angle of 78° when LFP/C was modified with hydrophilic benzene-trifluoromethanesulfonylimide groups [33]. This suggested that the electrode fabrication method did not alter the modified material.

### 3.3. Electrochemical Performance of LFP/LTO Batteries

An important step of our study was to validate through basic electrochemical tests in coin-cells that our modification method did not alter the performance of the electrode material. The electrochemical performances of the unmodified and modified LFP/C electrodes were evaluated in LiFePO_4_/Li_4_Ti_5_O_12_ full cells. A full cell consisting of a Li_4_Ti_5_O_12_ anode and a LiFePO_4_ cathode can provide a voltage of approximately 2 V, and the reaction occurs as follows:

3 LiFePO_4_ + Li_4_Ti_5_O_12_↔ 3 FePO_4_ + Li_7_Ti_5_O_12_ (1)

Li_4_Ti_5_O_12_ can accept three Li ions according to Equation (2) and shows a theoretical capacity of 175 mAh.g^-1^ [51]:

Li_4_Ti_5_O_12_ + 3 Li^+^ + 3 e^-^ → Li_7_Ti_5_O_12_ (2)

The theoretical capacity of LiFePO_4_ is 170 mAh·g^−1^, and the electrochemical reaction involves one Li ion. LiFePO_4_ should be present in excess in comparison to the titanate anode material (three times more) for the optimization of this system following Equation (1).

**Figure 5:**
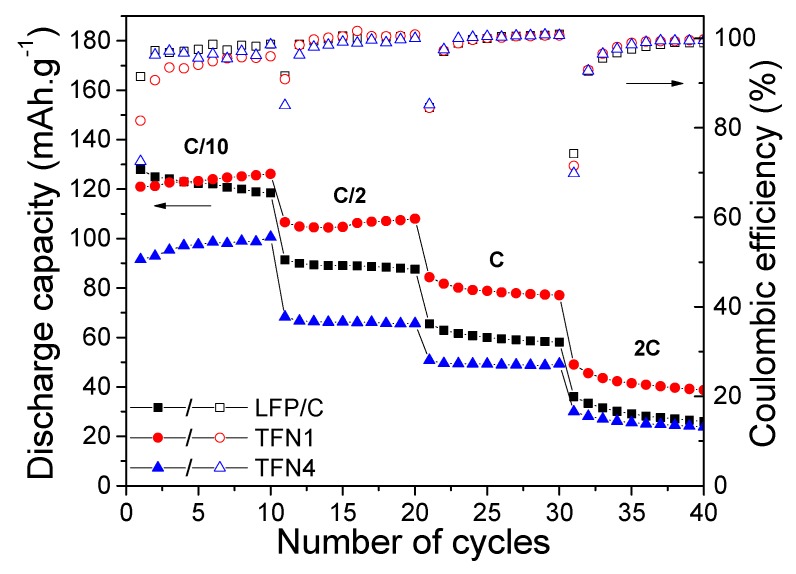
Discharge capacities (solid symbols) and Coulombic efficiencies (hollow symbols) of unmodified (-■/□-) and modified LFP/C cathodes, TFN1 (-●/○-), and TFN4 (-▲/∆-), cycled versus a Li_4_Ti_5_O_12_ (LTO) anode at different C-rates.

The main purpose of this work was to demonstrate the enhanced stability of LiFePO_4_ in the presence of air and water. Consequently, the electrochemical performance reported here could be improved by optimization of the formulation of the composite electrode, using the LTO anode coated with a carbon layer, calendering the electrodes, performing formation cycles at a low C-rate, and optimizing the pressure between electrodes. Since, in our cells, Li_4_Ti_5_O_12_ was slightly in excess, the discharge capacities for the modified and unmodified LFP/C cathodes (Figure 5) were not as high as expected because of the consumption of lithium during the first cycle. The grafting reaction, which led to the partial oxidation of the LFP/C powder, could probably have been one of the reasons for the low discharge capacity of the TFN4 electrode at C/10 (95 mAh·g^−1^). Since 30% of lithium was lost during the grafting reaction, the theoretical capacity of LFP could not be achieved even with the appropriate balancing of the electrodes. In addition, the relatively high loading of trifluoromethylphenyl groups for TFN4 could explain the rapid degradation of its electrochemical performance even at low C-rates [32]. The TFN1 electrode with the lowest grafted group loading delivered a discharge capacity of 125 mAh·g^−1^ at C/10, which was comparable to that of the unmodified LFP/C electrode. However, at higher cycling rates, the TFN1 electrode showed a slightly better rate performance than the unmodified LFP/C electrode. A similar observation has been reported for bromophenyl-modified LFP/C electrodes. The electrochemical performance of such electrodes deteriorates with an increase in the modifier loading from 0.4 to 1.1 wt.% [32]. Similarly, benzene-trifluoromethylsulfonimide-modified LFP/C with a grafted group loading of 2.1 wt.% has been reported to retain 46.1% of its initial discharge capacity (C/10) at 5C, while unmodified LFP/C retains only 14.8% of its initial discharge capacity [33].

**Figure 6:**
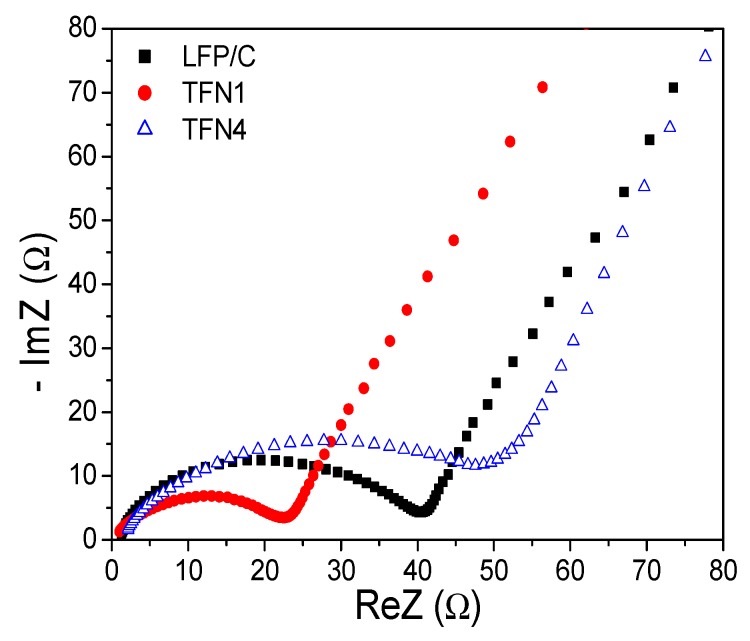
EIS results for the unmodified (-■-) and modified LFP/C cathodes, TFN1 (-●-) and TFN4 (-∆-), in a full cell with the same LTO anode (see the Experimental Section).

The Nyquist plots of the electrodes are shown in Figure 6. The plots showed an intercept on the real axis at high frequencies attributed to the resistance of the electrolyte. A semicircle was observed in the high-middle frequency region for all electrodes. The diameters of these semicircles on the Z_re_ axis were approximately equal to the charge-transfer resistance through the electrode/electrolyte interface [52]. The 45° straight line in the low frequency region could be attributed to the diffusion of lithium ions into the bulk of the electrode [53]. Since the same LTO anode was used for all the batteries assembled, hence the difference of impedance was ascribed to the grafting. The TFN4 electrode showed the highest charge transfer resistance (55 Ω), and hence the lowest discharge capacity among the three electrodes. Moreover, its semicircle was somewhat flattened probably because of the contribution of a second semicircle associated with the grafted layer. On the other hand, the TFN1 cathode showed considerably lower charge transfer resistance (23 Ω) than the unmodified LFP/C electrode (41 Ω). This was also consistent with the slightly superior rate capability of the TFN1 electrode (Figure 5) to that of the unmodified TFN1 electrode. A similar behavior has been reported for bromophenyl- [32] and benzene-trifluoromethylsulfonimide-modified LFP/C electrodes [33].

### 3.4. Chemical Lithiation of the Oxidized Samples

**Figure 7:**
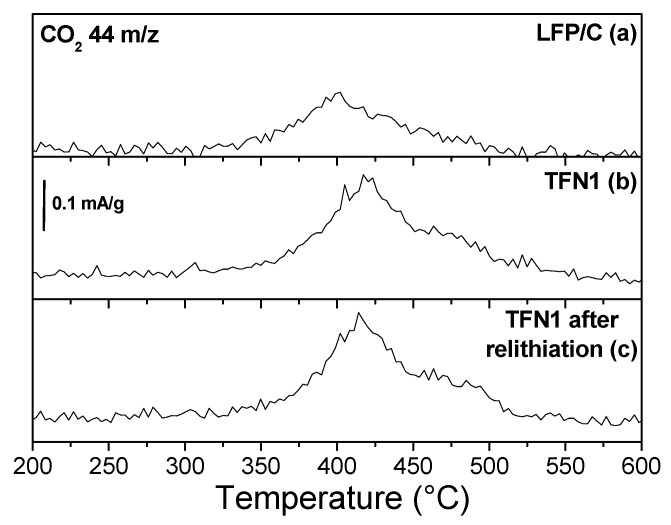
Mass spectra recorded during the thermogravimetric analysis of (**a**) LFP/C and modified LFP/C (TFN1), (**b**) before and (**c**) after chemical relithiation. The m/z ratio of 44 corresponds to the CO_2_ formed during the decomposition of the powder.

Since the grafting of LFP/C with trifluoromethylphenyl groups involved its delithiation, the possible lithiation mechanism of the modified samples was investigated. The chemical lithiation of the modified LFP/C powders was carried out using LiI as the reducing agent and lithium source [54]. First, the stability of the grafted layer during the chemical lithiation process was investigated by carrying out the TGA/MS analyses of the relithiated powders. The mass spectra of unmodified LFP/C (showing CO_2_ evolution) and grafted TFN1 sample before and after the relithiation process are shown in Figure 7.

**Figure 8:**
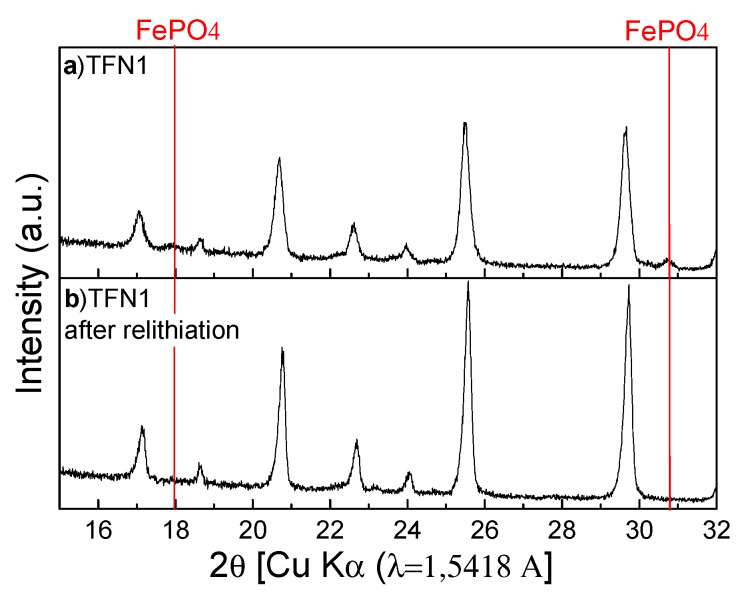
XRD patterns for TFN1 (**a**) before and (**b**) after relithiation.

In the case of the TFN1 powder, the onset of CO_2_ evolution occurred at the same temperature before and after the chemical lithiation process. This temperature was lower than the CO_2_ evolution onset temperature of unmodified LFP/C. Moreover, the intensity of the CO_2_ peak for LFP/C was weaker than those for the modified samples. These results can be attributed to the large carbon content of the groups grafted on LFP/C [33]. In addition, the modified sample showed similar MS spectra before and after the chemical relithiation process. The second CO_2_ contribution at 450–550 °C for the two TFN1 samples (Figure 7b,c) could be attributed to the presence of the grafted groups. These results clearly demonstrated that the chemical lithiation did not affect the grafted layer because of the similarity of the CO_2_ signal. The XRD pattern of the relithiated TFN1 is shown in Figure 8b. After the chemical relithiation process, the FePO_4_ peaks observed before chemical relithiation (Figure 8a) disappeared completely, while those corresponding to LiFePO_4_ became more intense.

## 4. Conclusions

The aim of this work was to demonstrate the facile protection of an LFP/C powder from side-reactions (e.g., lithium loss) occurring in contact with water. We demonstrated that the functionalization of LFP/C with trifluoromethylphenyl groups by diazonium chemistry is an efficient strategy to control its surface reactivity. The stability of the LFP/C powder in water increased considerably after the modification. Therefore, this surface modification approach rendered LFP/C suitable for various applications. For example, air-stable materials would be cost-effective for storage and shipment. The use of water-soluble binders (e.g., carboxymethylcellulose sodium salt) and water as a solvent for ink formation would be possible with a material stable in water and would promote the fabrication of green batteries [55]. This work stands out from other studies dealing with the water slurry processing, which usually report the effects of ink preparation [56] or the use of surfactants [57] on the electrochemical performance of Li-ion batteries. In fact, while the use of surfactants or high shear techniques are of interest for improving the dispersion of electrode materials in the ink, they did not improve the stability of the cathode material in contact for several hours in water. In addition, this modification technique can be particularly adapted for aqueous rechargeable lithium batteries, which suffer from the dissolution of the electrode material or its oxidation because of its reactivity with aqueous electrolytes [58]. Furthermore, this modification method can be applied to other carbon-coated materials and especially to lithium nickel manganese cobalt oxide (NMC) powders, which suffer from severe degradation in contact with water [59]. The electrochemical performance of the modified LFP/C electrode was not adversely affected by the presence of trifluoromethylphenyl groups at the carbon surface despite the formation of a delithiated LFP material. Thus, the modified LFP/C material (especially TFN1) could be used in a full Li-ion battery. Furthermore, the chemical relithiation of the trifluoromethylphenyl-modified powder was demonstrated. This approach can be used to prepare cathode materials that are both grafted and totally lithiated. The grafting process reported in this study can be potentially improved by using fewer precursors, especially tert-butyl nitrite, in order to avoid the oxidation of the material. The utilization of the corresponding diazonium salt (ex. CF_3_-phenyl-N_2_^+^BF_4_^−^) could lead to a more efficient grafting reaction [34].

## Figures and Tables

**Table 1:** Grafted group loading (wt. %) and the amount x of lithium deinserted, as estimated using the TGA and ICP-AES results for the unmodified powder (LFP/C) and the grafted samples (TFN1 to TFN4).

Sample	Amine (mmol)	Tert-butyl Nitrite (mmol)	wt.% of Grafted Groups (TGA)	x in Li_(1-x)_FePO_4_ by TGA	x in Li_(1-x)_FePO_4_ by ICP
LFP/C	-	-	-	0	~ 0
TFN1	2	6	0.55	0.20	0.20
TFN2	4	12	0.60	0.27	0.25
TFN3	6	18	0.71	0.26	0.27
TFN4	8	24	0.78	0.32	0.28

**Table 2:** Atomic percentage of different elements determined by XPS for the modified (TFN1 and TFN4) and unmodified LFP/C powders and reagent concentrations used for the modification.

Sample			Atomic Concentration (%)					
	Amine (mmol)	Tert-butyl Nitrite (mmol)	C 1s	O 1s	N 1s	P 2p	Fe 2p	F 1s
LFP/C	-	-	42.5	41.6	0.2	10.4	5.3	0.0
TFN1	2	6	38.0	43.3	0.5	9.5	5.7	3.0
TFN4	8	24	43.9	37.5	0.6	8.2	4.3	5.5

**Table 3:** Oxidation levels of unmodified and modified LFP/C (TFN4) powders for different reaction conditions in water, as determined using ICP-AES.

Sample-Conditions	x in Li_(1-x)_FePO_4_ by ICP *	Δx
LFP/C	~ 0	-
LFP/C–24 h of immersion, ambient temperature	0.34	0.34
LFP/C–18 h of immersion, 60 ˚C	0.29	0.29
TFN4	0.28	-
TFN4–24 h of immersion, ambient temperature	0.30	0.02
TFN4–18 h of immersion, 60 ˚C	0.32	0.04

* “x” refers to the fraction of lithium deinserted from LFP structure after surface modification or immersion in water.

## Supplementary Materials

The following are available online at https://www.mdpi.com/1996-1944/13/4/942/s1, Figure S1: XPS survey spectra for the modified (TFN1 and TFN4) and unmodified LFP/C powders. The red dotted rectangle shows the fluorine signal (F 1s peaks). Table S1: Concentration of iron dissolved in the 1M LiPF6 EC:DC:DMC (1:1:1) electrolyte for modified and unmodified LFP/C after one month of immersion.
