# Supplementary material for: Protection of LiFePO4 against Moisture
Source: Materials (Basel). 2020 Feb 20;13(4):942. doi: 10.3390/ma13040942 (PMC7078597; doi:10.3390/ma13040942)
Supplement: Supplementary file 1 [file materials-13-00942-s001.pdf]

materials

MDPI

*Article*

# Protection of $LiFePO_4$ Against Moisture

**Nicolas Delaporte [1], Michel L. Trudeau [2], Daniel Bélanger [1,*] and Karim Zaghib [2,*]**

[1] Département de Chimie, Université du Québec à Montréal, Case Postale 8888, succursale Centre-Ville, Montréal (Québec) Canada H3C 3P8; delaporte.nicolas@hydro.qc.ca

[2] Institut de Recherche d'Hydro-Québec (IREQ), 1800 Boulevard Lionel Boulet, Varennes, QC, Canada J3X 1S1; trudeau.michel@hydro.qc.ca

* Correspondence: zaghib.karim@hydro.qc.ca (K.Z.); belanger.daniel@uqam.ca (D.B.)

SI1) X-ray photoelectron spectroscopy (XPS)

The XPS survey spectra for the modified and pristine LFP/C powders are shown in Figure SI1. Compared to the pristine LFP/C powder, the TFN1 and TFN4 samples showed an additional F 1s peak around 687 eV because of the presence of trifluoromethylphenyl groups on their carbon surfaces. Furthermore, the weak P2p and P2s peaks at 135 and 192 eV for the modified powders as compared to those of LFP/C confirms the presence of a surface layer.

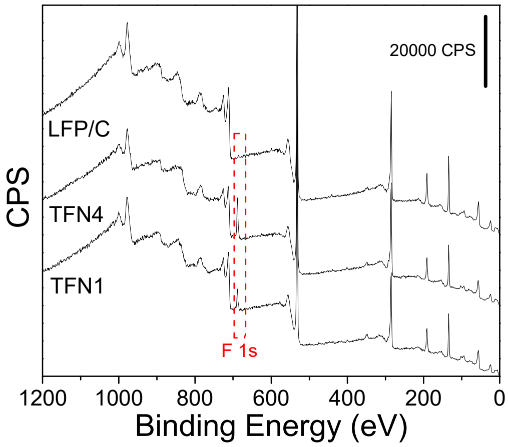


**Figure S1.** XPS survey spectra for the modified (TFN1 and TFN4) and unmodified LFP/C powders. The red dotted rectangle shows the fluorine signal (F 1s peaks).

SI2) Evaluation of iron dissolution in the electrolyte

Based on the active material/volume of the electrolyte ratio used in the battery, a simple experiment was carried out to quantify the iron dissolution of the unmodified and modified LFP/C electrodes in the electrolyte. Table SI1 lists the concentrations of the ion dissolved in the electrolyte (ppm) for the unmodified and modified LFP/C electrodes after 1 month of immersion. The addition of the new organic layer also reduced the iron dissolution of the modified electrode in the electrolyte. The modified and the unmodified LFP/C electrodes showed an iron dissolution of 4.6 and 9.1%, respectively in the electrolyte.

**Table S1.** Concentration of iron dissolved in the 1M $LiPF_6$ EC:DC:DMC (1:1:1) electrolyte for modified and unmodified LFP/C after one month of immersion.

| Sample | Iron concentration in the electrolyte after 1 month of immersion (ppm) |
|---|---|
| LFP/C | 13 |
| TFN4 | 6 |
